# Sinus Tachycardia and Unrelieved Wall Stress Precede Left Ventricular Systolic Dysfunction During Preclinical Cardiomyopathic Changes in Duchenne Muscular Dystrophy

**DOI:** 10.3390/jcdd12080280

**Published:** 2025-07-23

**Authors:** Takeshi Tsuda, Amy Walczak, Karen O’Neil

**Affiliations:** 1 Nemours Cardiac Center, Nemours Children’s Health Delaware, 1600 Rockland Rd., Wilmington, DE 19803, USA; 2 Department of Pediatrics, Sidney Kimmel Medical College at Thomas Jefferson University, Philadelphia, PA 19107, USA

**Keywords:** muscular dystrophy, wall stress, cardiomyopathy, echocardiography, dystrophin, mechanosensor

## Abstract

Background: The onset of cardiomyopathy in Duchenne muscular dystrophy (DMD) is insidious and poorly defined. We proposed integrated wall stress (iWS) as a marker of total left ventricular (LV) workload and tested whether the increased iWS represents early DMD cardiomyopathy. Methods: Peak systolic wall stress (PS-WS) was calculated in M-mode echocardiography with simultaneous blood pressure measurement. iWS was defined as a product of PS-WS and heart rate (HR) divided by 60 (=PS-WS/RR interval). We measured iWS in normal controls (CTRL), DMD with normal LV shortening fraction (%LVSF ≥ 30%) (DMD-A), and DMD with decreased %LVSF (<30%) (DMD-B). Results: 40 CTRL and 79 DMD patients were studied. Despite comparable %LVSF, both HR and iWS were significantly higher in DMD-A (n = 50) than in CTRL (*p* < 0.0001). iWS was significantly higher in DMD-B (n = 29) than in DMD-A (*p* < 0.0001) despite comparable HR. PS-WS was significantly higher in DMD-A than in CTRL and higher in DMD-B than in DMD-A, suggesting high HR is not a sole determinant of increased iWS in DMD-A compared with CTRL. In a longitudinal study in 35 DMD patients over 4.0 ± 2.0 years, iWS showed significant increase (*p* = 0.0062) alongside a significant decline in %LVSF (*p* < 0.0001). Conclusions: iWS significantly increased in DMD before %LVSF declined. The progressive increase of iWS in DMD is initially associated with increased HR and then with increased PS-WS. iWS may serve as a useful echocardiographic marker in identifying preclinical DMD cardiomyopathy.

## 1. Introduction

Cardiomyopathy is a common complication in Duchenne muscular dystrophy (DMD) seen in mid-teens [1,2], but its onset is insidious and poorly characterized [3]. Due to progressive systemic muscle weakness and inability to voluntary movement, DMD patients often do not report common symptoms of exercise intolerance. Since progressive LV dysfunction, cardiomyopathy, and heart failure are inevitable in DMD, mostly by their late teens, it is imperative to detect early myopathic changes for prompt treatment.

Echocardiography is widely used as a surveillance study in outpatient clinics, but conventional measurement of LV systolic function and geometry does not serve as a sensitive marker to address early preclinical myopathic changes. Certain echocardiographic approaches are proposed to assess the development of early myopathic changes in DMD hearts, especially diastolic function [4,5], tissue Doppler imaging and myocardial performance index [6], as well as LV strain [7,8]. However, the reliability of echocardiographic assessment of LV diastolic function in children has been controversial [9], and the efficacy of the new technology-based methods has certain limitations, including poor acoustic windows, reproducibility of measurements, and inter-vendor differences [10].

We previously proposed a novel concept of integrated wall stress (iWS) to quantitatively measure total LV workload in the mouse model by simultaneous LV pressure measurement by left heart catheterization and M-mode echocardiography [11]. iWS was defined as cumulative LV wall stress throughout the cardiac cycle over one minute, representing a more reliable quantification of continuous LV workload. iWS encompasses ventricular wall stress, contractile status, and HR, all of which determine myocardial oxygen consumption [12]. A strong correlation between iWS (equivalent to average WS throughout a cardiac cycle) and a product of systolic WS and HR was noted (R = 0.70), postulating that this product may serve as a feasible non-invasive marker for LV workload [11].

Here, we investigate whether iWS, defined as a product of PS-WS and HR obtained non-invasively by M-mode echocardiography and simultaneous blood pressure (BP) measurement, serves as a reliable marker to detect early myopathic changes in DMD hearts. We hypothesized that increased iWS precedes LV dysfunction in the preclinical phase of DMD cardiomyopathy.

## 2. Patients and Methods

### 2.1. Patients

We performed retrospective, cross-sectional, and longitudinal studies to assess LV systolic performance by M-mode echocardiography and simultaneous HR and BP measurements in healthy control patients without heart disease (CTRL) and DMD patients in compliance with a protocol approved by the Institutional Review Board (IRB) of Nemours Children’s Health Delaware. CTRL consisted of randomly enrolled males who underwent routine echocardiograms for certain nonspecific symptoms (chest pain, palpitations, or dizziness), functional murmur, or minor ECG abnormalities (first-degree atrioventricular block or premature ventricular contractions) with normal cardiac anatomy and function and who underwent simultaneous BP measurements at echocardiographic measurements. For DMD patients, cardiac medications were also examined, including angiogenin II converting enzyme inhibitors (ACEI), angiotensin II receptor blockers (ARB), β-blockades (BB), and anti-aldosterone agents. Echocardiograms are performed as a standard outpatient procedure. The studies were performed in the Echocardiography Laboratory at the Nemours Cardiac Center, Nemours Children’s Health Delaware.

### 2.2. Echocardiographic Analysis

Routine 2D-echocardiography was performed by experienced certified sonographers for pediatric/congenital heart disease (iE33 xMATRIX Echocardiography System; Philips, Andover, MA, USA). M-mode measurements were obtained by parasternal short axis view at the level of papillary muscles in a supine position as a part of our routine pediatric echocardiographic assessment. Interventricular septal thickness in systole (IVSs), LV internal diameter in systole (LVIDs), LV posterior wall thickness in systole (LVPWs), and %LV shortening fraction (%LVSF) were measured. LV wall thickness (LVWTs) was defined as an average of IVSs and LVPWs. Systolic blood pressure (SBP) was measured simultaneously with the M-mode study. M-mode measurements of LV dimensions and simultaneous BP were repeated 3 times in each patient. All presented values were the average of three individual measurements.

### 2.3. Grouping of the DMD Patients

DMD patients were divided into (i) %LVSF ≥ 30% (DMD-A) and (ii) %LVSF < 30% (DMD-B). DMD-A patients were further sub-grouped into two age groups; <10 years old (yo) and ≥10 yo. For the longitudinal study, we selected the earliest and the latest echocardiograms during the study period, as most DMD patients had multiple studies.

### 2.4. iWS

The concept of iWS was originally introduced in the mouse model with continuous LV pressure measurement by cardiac catheterization and simultaneous LV diameter measurement by M-mode echocardiography to calculate cumulative LVWS (integration of LVWS over time) as a measurable marker for LV workload, where a good correlation was demonstrated between iWS and PS-WS × HR [11]. PS-WS can be calculated noninvasively according to original Laplace’s law.

PS-WS = P·R*_i_*^2^/[(R*_o_* − R*_i_*)(R*_o_* + R*_i_*)] = P·R*_i_*/2h(1 + h/2R*_i_*)                   = [SBP·LVIDs]/[2LVWTs˒(1 + LVWTs/2LVIDs)](1)

P = pressure (SBP), R*_o_* = outer diameter, R*_i_* = inner diameter (LVIDs), h = wall thickness (LVWTs).

In a simpler version, PS-WS is also estimated by multiplying SBP and LV diameter at peak systole (LVIDs) and dividing by LV wall thickness at peak-systole (LVWTs).

PS-WS = SBP·LVIDs/LVWTs (2)

Here, we define iWS as cumulative LV WS over one second instead of one minute, as shown in the original study.

iWS = (PS-WS × HR)/60                  = PS-WS/RR interval                                       = SBP·LVIDs/(LVWTs·RR interval)(3)

### 2.5. Statistics

The data are shown as means ± standard deviation (SD) for continuous variables, unless otherwise noted. A two-sample *t*-test was used to compare the means and proportions between the two groups. Multiple comparisons (more than 2 groups) were performed by one-way analysis of variance (ANOVA), followed by Tukey’s multiple comparison test to assess the significance of data values. Linear regression analysis was performed to assess the strength and direction of the linear relationship between the two variables. Analysis of covariance (ANCOVA) was used to compare the regression lines between two groups. Statistics were assessed by GraphPad Prism 5 (GraphPad, San Diego, CA, USA). A *p* value of less than 0.05 is regarded as statistically significant.

## 3. Results

### 3.1. Patients and Echocardiographic Findings

We studied 40 CTRL and 79 DMD patients from January 2011 to December 2018. The DMD patients consisted of 50 DMD-A (normal %LVSF) and 29 DMD-B (decreased %LVSF). DMD-A were further divided into 21 DMD-A (<10 yo) and 29 DMD-A (≥10 yo). For the cross-sectional study, we chose the first echocardiogram during the enrollment period.

Age, HR, SBP, M-mode echocardiographic measurements, and medications are summarized in Table 1. DMD-A (<10 yo) is significantly younger, while DMD-B is significantly older than the rest of the groups: CTRL and DMD-A (≥10 yo) are of comparable ages. HR is significantly higher in all DMD groups than in CTRL but is comparable among all DMD groups. SBP is significantly lower only in DMD-A (<10 years) compared with the rest of the groups. %LVSF is significantly lower only in DMD-B compared with the rest but shows no difference between CTRL, DMD-A (<10 years), and DMD-A (≥10 years). LVIDs is significantly lower only in DMD-A (<10 yo) and significantly higher only in DMD-B compared with the rest. Systolic LV wall thickness (LVWTs) is significantly lower in all DMD groups compared with CTRL.

Medications taken by DMD patients included angiotensin II converting enzyme inhibitors (ACEI; enalapril or lisinopril), angiotensin II receptor blockers (ARB; losartan), β-blockers (BB; carvedilol or metoprolol), anti-aldosterone agents (spironolactone or eplerenone), and corticosteroids (prednisone or deflazacort). Overall, medications were more frequently prescribed in older DMD patients with decreased %LVSF. In particular, BB was more frequently prescribed in DMD-B, which may be influencing HR.

**Figure 1:**
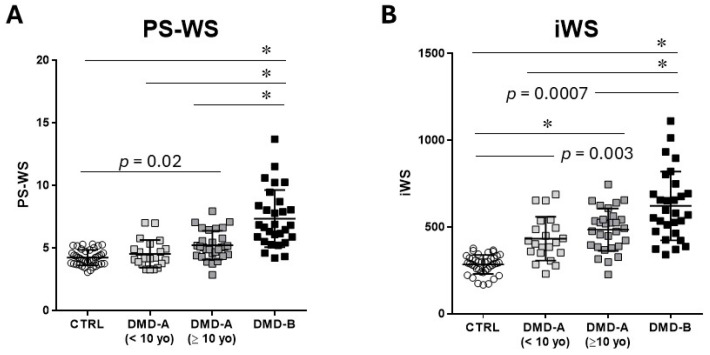
**PS-WS and iWS.** PS-WS and iWS were presented in control (CTRL), DMD-A (<10 yo), DMD-A (≥10 yo), and DMD-B. (**A**): PS-WS in DMD-A (≥10 yo) is significantly higher than CTRL, whereas there is no significant difference between DMD-A (<10 yo) and control. (**B**): iWS in DMD-A is significantly higher than that in CTRL. DMD-B exhibits significantly higher PS-WS and iWS than the others. * *p* < 0.0001.

We compared the PS-WS values obtained by an original form (1) and that by a simplified formula (2) in the Method section. There was an excellent correlation between the two methods (y = 0.226x + 4.33, R = 0.997). Thus, we calculated PS-WS only by a simplified formula in this study. PS-WS is significantly elevated in DMD-A (≥10 years) (5.19 ± 1.02) compared with CTRL (4.20 ± 0.61) and is significantly higher in DMD-B (7.87 ± 2.31) than in the other groups; there is no significant difference between CTRL and DMD-A (<10 yo) (4.85 ± 1.01) (Figure 1A).

iWS is significantly elevated in all DMD groups compared with CTRL (283 ± 56) and is significantly higher in DMD-B (686 ± 213) than in DMD-A groups: no significant difference is noted between DMD-A (<10 yo) (444 ± 123) and DMD-A (≥10 yo) (483 ± 132) (Figure 1B). Notably, both DMD-A groups exhibit significantly higher iWS than CTRL despite comparable %LVSF. Both PS-WS and iWS are comparable between DMD-A (<10 yo) and DMD-A (≥10 yo).

### 3.2. Relationship Between iWS and HR

**Figure 2:**
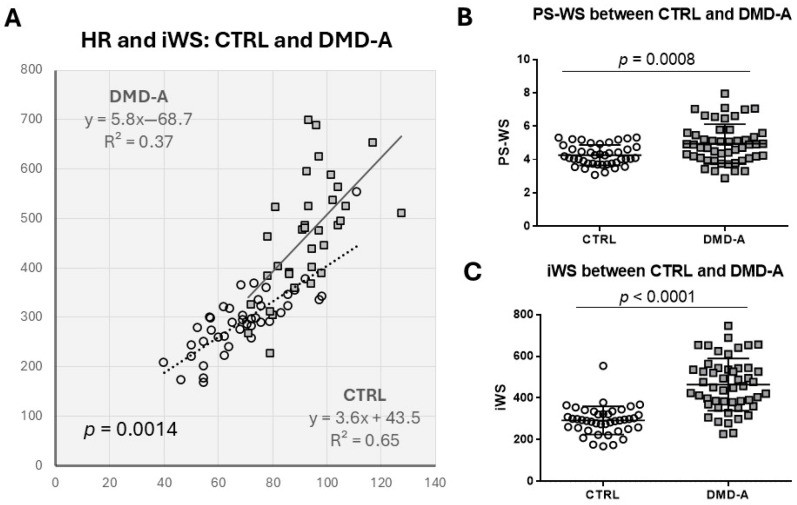
**The relationship between HR and iWS in DMD-A.** (**A**): There is a very good correlation between HR and iWS in both CTRL and DMD-A. The difference in slope of two regression lines is significant (*p* = 0.0014) by ANCOVA, suggesting HR is not the only determinant of iWS in DMD-A. (**B**,**C**): Significant differences are shown in PS-WS and iWS between CTRL and DMD-A.

As iWS is defined as a product of PS-WS and HR, we investigated whether the increase of iWS in DMD patients is predominantly attributed to the increase in HR. In the following analysis, we combined two DMD-A groups into one to assess the difference between CTRL and DMD-A (=DMD with normal LV systolic function), as there is no notable difference in PS-WS or iWS between the two DMD-A groups. HR and iWS show a very good positive linear correlation in both CTRL and DMD-A, more in CTRL (R = 0.81) than in DMD-A (R = 0.61) (Figure 2A). The slope of the regression line of DMD-A is steeper than that of CTRL (*p* = 0.0014), suggesting HR is not a sole determinant of iWS in DMD-A. When PS-WS and iWS are compared between NL and DMD-A, DMD-A reveals significantly higher PS-WS and iWS than NL despite comparable %LVSF, indicating both increased PS-WS and HR are responsible for the increased iWS in DMD-A compared with CTRL (Figure 2B,C). Both PS-WS and iWS are significantly higher in DMD-B compared with DMD-A (<10 yo) and DMD-A (≥10 yo) (Figure 1), suggesting both HR and PS-WS increases likely contribute to the progression of LV dysfunction.

### 3.3. Longitudinal Study of LV Dysfunction of DMD

**Figure 3:**
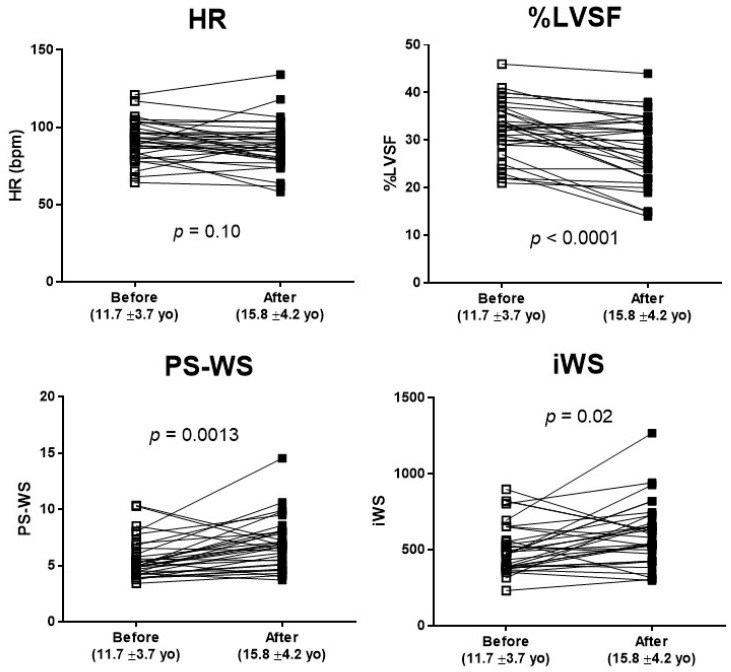
**Longitudinal changes in HR, %LVSF, PS-WS, and iWS in DMD patients.** Echocardiographic changes in 35 DMD patients who had multiple echocardiograms during the study period of 4.1 ± 2.0 years. Although HR remained relatively unchanged, there was a significant decline in LVSF (*p* < 0.0001) in line with a significant increase in PS-WS and iWS as DMD patients grew.

Total 35 DMD patients who had multiple echocardiograms were examined longitudinally over two time points, at the earliest (11.7 ± 3.7 yo) and the latest encounters (15.8 ± 4.2 yo) during the study period, with an interval duration of 4.1 ± 2.0 years (Figure 3). Over this time period, %LVSF was significantly reduced from 32.3 ± 6.0% to 28.3 ± 7.3%, whereas PS-WS and WSI significantly increased during this time (5.55 ± 1.76 to 6.86 ± 2.27 and 507 ± 163 to 600 ± 205, respectively). HR showed no significant change during this period.

## 4. Discussion

We have demonstrated that iWS is significantly increased before %LVSF declines in DMD patients. In an early preclinical phase, DMD-A (<10 yo), the increase in iWS is mainly attributed to increased HR, whereas the increase in PS-WS is a predominant factor in increasing iWS in the later stages, DMD-A (≥10 yo) and DMD-B. The longitudinal observation reveals that iWS increases as LV systolic dysfunction exacerbates without significant HR increase, suggesting unrelieved WS is a predominant determinant for the development of DMD cardiomyopathy in the later stage. Here, we propose iWS as a useful echocardiographic marker to quantify the degree of mechanical stress of LV myocardium that precedes LV dysfunction and abnormal LV geometric changes during the early pathological remodeling, first by an increase in HR and then by increase in PS-WS.

### 4.1. Early Detection of the Preclinical Stage of DMD Cardiomyopathy

DMD patients are known to develop cardiomyopathy and heart failure after their mid-teen years, but the detection of early myopathic transition is unclear because of its insidious onset and lack of symptoms due to patients’ limited voluntary physical activities [13,14]. Diagnosis of the preclinical stage of cardiomyopathy in DMD patients before the emergence of LV dysfunction is essential in managing these patients, as early treatment may provide beneficial effects in attenuating the development of cardiomyopathy and LV dysfunction [15]. Several new echocardiographic techniques have been proposed to address early myopathic changes in DMD hearts that precede the decline in global LV systolic function, as described earlier [6,7,8,16,17]. These approaches are based on the hypothesis that the presence of preclinical focal myocardial damage or myocardial distortion precedes the development of global LV systolic dysfunction. On the other hand, DMD cardiomyopathy should be characterized by an absence of dystrophin, which is distinct from other forms of dilated cardiomyopathies (DCM) [16,18,19].

The principal pathogenesis of DMD cardiomyopathy consists of progressive myocyte loss (atrophy) and concomitant tachycardia [20], both of which increase ventricular WS and overall ventricular workload. Myocardial fibrosis is a secondary phenomenon following cardiomyocyte death, which presents in a variable timing and a heterogeneous fashion, primarily detected by cardiac MRI [21]. Variable degrees of ventricular dilatation may occur only in an advanced stage, not in an early stage, of DMD cardiomyopathy.

In our DMD patients, we demonstrated that the earliest signs of myopathic change are an increase of iWS and persistent sinus tachycardia, as seen in DMD-A (<10 yo) when compared with CTRL (Figure 1). The mechanisms for persistent sinus tachycardia in early DMD cardiomyopathy remain undetermined but may be due to autonomic dysfunction [22], decline in myocardial compliance [4], or regional myocardial abnormalities [8,17]. Persistent tachycardia certainly contributes to the increase of iWS in a preclinical stage. However, increased HR is not the sole reason for an increase in iWS, as PS-WS was also increased during the early phase, DMD-A (≥10 yo) (Figure 3). Tachycardia results in increased LV workload and thus increases myocardial oxygen demand [23], which is represented by the increase in iWS. Independently, increased LVIDs with thinner LVWTs in DMD-B further raise PS-WS. These combined negative impacts may contribute to further pathological ventricular remodeling in DMD patients.

### 4.2. Physiological Significance of WS in Understanding Cardiac Remodeling

Systolic WS not only represents the ventricular afterload against which the myocardium must work to pump out the blood but it also elucidates the degree of myocardial deformation reflecting a combination of primary disease processes and secondary compensatory responses [24]. In addition, ventricular WS is one of the primary determinants of myocardial oxygen consumption [23,25], and normalization of WS is considered a compensatory response to minimize excessive workload on the ventricular myocardium [24]. Failure in normalizing WS may result in unresolved myocardial workload, inducing a progressive maladaptive sequence of ventricular remodeling where interactions between WS, geometric changes, and energetic outcomes play a central role [26].

The concept of iWS was introduced to measure the entire workload throughout one cardiac cycle sensed by the LV myocardium and was proposed as a more reliable marker for total LV workload than a one-point measurement of systolic WS [11]. As WS changes during the cardiac cycle, where diastolic WS was found to be negligible compared with the PS-WS in humans [27], we proposed iWS (PS-WS × HR) as a reasonably reliable noninvasive clinical marker to estimate total LV workload [11]. Similarly, Devereux et al. introduced the concept of the LV wall stress-mass-heart rate product (triple product) in hypertensive patients as a clinical marker for myocardial oxygen demand and demonstrated that the patients with eccentric LV hypertrophy showed higher triple products than those with control or concentric hypertrophy [28]. In 1522 asymptomatic adult patients with mild to moderate aortic stenosis (AS), a higher triple product was shown to be associated with higher cardiovascular morbidity and mortality, suggesting higher myocardial oxygen demand is contributing to the increased adverse event rate in AS patients [29]. Similarly, in our DMD patients, the increased iWS represents increased myocardial oxygen demand in structurally vulnerable DMD myocardium, further aggravating myopathic changes.

### 4.3. Unique Pathophysiology of DMD Cardiomyopathy 

Dystrophic hearts are abnormally vulnerable to increased biomechanical stress, as demonstrated in the dystrophin-deficient mouse model (mdx mice) [30]. Vatta et al. demonstrated that dystrophin expression in the ventricular myocardium was markedly diminished in advanced human heart failure, but it recovered significantly after the reduction of mechanical stress by a ventricular assist device (VAD) in combination with the recovery of ventricular function, suggesting that dystrophin might provide a final common pathway in determining myocardial contractile reserve [31]. DMD patients lack this critical function. Thus, measuring the degree of LV workload may help predict the prognosis of LV myocardium in DMD patients. Insufficient thickening of the LV wall during peak systole may be due to a lack of normal growth response or compensatory hypertrophy in DMD patients, as the dystrophin-glycoprotein complex serves as one of the critical mechanosensors of muscle cells [32]. In the mouse model, a mechanosensing role of the dystrophin-glycoprotein complex was shown to mediate cardiomyocyte growth via activating AMP-activated protein kinase (AMPK), which induces neuronal nitric oxide synthetase (nNOS) [33]. The absence of dystrophin may perturb an ordinary compensatory response to biomechanical stress. DMD heart may be uniquely characterized by an inability to normalize WS with progressive myocyte loss and subsequent myocardial fibrosis.

Disproportional resting sinus tachycardia is another feature of DMD cardiomyopathy [20]. The impact of tachycardia may be even greater due to reduced HR variability, resulting in higher average HR in DMD patients [34]. As tachycardia increases iWS, early treatment with β-blockers may be beneficial in attenuating myopathic changes in DMD hearts by reducing LV workload, even before %LVSF decreases. The pharmacologic effects of β-blockers include reducing HR and BP, resulting in decreased total LV workload. As β-blockers have been proven beneficial in attenuating or improving LV dysfunction in advanced-stage DMD patients [35], their early introduction in addition to angiotensin-converting enzyme inhibitors (ACEI) or angiotensin receptor blockers (ARB) may be therapeutically beneficial. Thomas et al. proposed the early introduction of β-blockers for DMD patients with persistent tachycardia to attenuate the progression of cardiomyopathy [20]. Our iWS findings in DMD patients support the rationale of this treatment.

**Figure 4:**
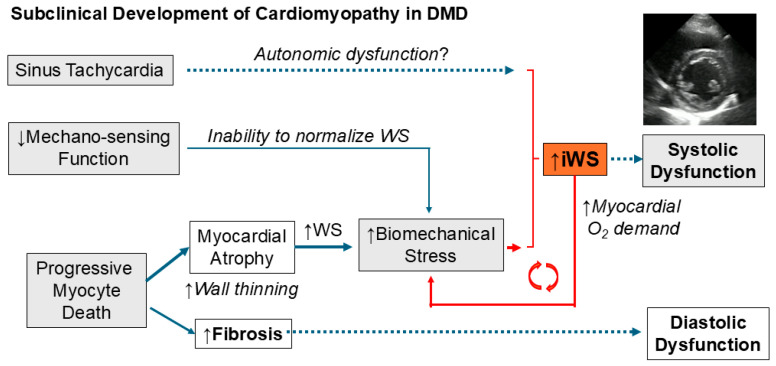
**Pathogenesis of DMD cardiomyopathy**. The subclinical phase of DMD cardiomyopathy is characterized by progressive cardiomyocyte death causing myocardial atrophy in combination with the inability to normalize WS due to diminished mechanosensing capacity and resting sinus tachycardia, resulting in a progressive increase in iWS.

The pathogenesis of early DMD cardiomyopathy is illustrated below (Figure 4).

### 4.4. Limitations

First, an early pathological process in DMD myocardium is likely to be more focal than global, as shown by increased abnormalities in myocardial strain pattern seen in DMD patients without overt cardiomyopathy [7]. iWS only represents global performance of LV myocardium, and our method may be oversimplifying the true pathological process in the early stage of DMD cardiomyopathy. A single M-mode measurement of LV diameter to represent LV volume obviously has inevitable limitations. We did not perform the regional assessment of cardiac deformation by other diagnostic modalities. Second, we only consistently measured %LVSF as a marker for LV systolic function and did not routinely obtain LV ejection fraction in all cases. Third, the study was not controlled for the medication of the patients. Some of our older cohort may not be concordant with the current treatment guidelines for DMD. More recent patients are receiving proactive cardioprotective medications at younger ages. Fourth, we did not specifically examine the inter-operator variability or repeatability. However, the M-mode measurements were performed by an echocardiographic sonographer with more than 20 years’ experience. Lastly, this is a retrospective study with a relatively small cohort in a single institution, which limits statistical power. A larger cohort study is warranted to support our current findings.

## 5. Conclusions

iWS can be obtained easily and non-invasively by using M-mode echocardiography and simultaneous BP measurement and is usually not affected by the poor acoustic windows. Increased peak systolic dimension, decreased peak systolic LV wall thickness, and increased heart rate all contribute to an increase in iWS. iWS can serve as a useful clinical marker in identifying early myopathic changes in DMD hearts when used with other advanced noninvasive imaging studies. 

## Figures and Tables

**Table 1:** Demographic and echocardiographic findings in control (CTRL) and Duchenne muscular dystrophy (DMD) patients DMD patients were divided into two groups based on left ventricular (LV) systolic function; DMD-A for LV shortening fraction (LVSF) ≥ 30% and DMD-B for LVSF < 30%. DMD-A was further divided at age 10 years; DMD-A (<10 yo) and DMD-A (≥10 yo). * *p* < 0.05 compared with CTRL, ^†^ *p* < 0.05 compared with DMD-A (<10 yo), ^‡^ *p* < 0.05 compared with DMD-A (≥10 yo).

	CTRL	DMD-A (<10 yo)	DMD-A (≥10 yo)	DMD-B
Number	40	21	29	29
Age (years)	13.2 ± 2.5	7.0 ± 1.7 *	13.7 ± 3.4 ^†^	15.4 ± 3.4 *^†‡^
HR (bpm)	68 ± 14	92 ± 14 *	93 ± 16 *	86 ± 12 *
SBP (mmHg)	103 ± 13	88 ± 13 *	104 ± 12 ^†^	101 ± 9 ^†^
**Echocardiogram**				
LVSF (%)	37.3 ± 3.4	35.8 ± 3.7	33.9 ± 4.8	24.6 ± 4.78 *^†‡^
LVIDs (cm)	3.04 ± 0.44	2.61 ± 0.29 *	2.93 ± 0.60	3.74 ± 0.64 *^†‡^
LVWTs (cm)	1.22 ± 0.22	0.83 ± 0.10 *	0.95 ± 0.14 *^†^	0.89 ± 0.19 *
**Medications**				
ACEI (%)	0 (0)	3 (14)	11 (38)	12 (41)
ARB (%)	0 (0)	0 (0)	8 (28)	12 (41)
BB (%)	0 (0)	0 (0)	2 (7)	11 (38)
Anti-Aldosterone (%)	0 (0)	0 (0)	2 (7)	1 (3)
Steroid (%)	0 (0)	7 (33)	14 (48)	10 (34)

HR: heart rate, SBP: systolic blood pressure, LVSF: left ventricular shortening fraction, LVIDs: left ventricular internal dimension in systole, LVWTs: average left ventricular wall thickness in systole, ACEI: angiotensin II converting enzyme inhibitor, ARB: angiotensin II receptor blocker, BB: β-blocker.

## Data Availability

The original contributions presented in this study are included in the article. Further inquiries can be directed to the corresponding author.

## Abbreviations

BP	Blood pressure
DMD	Duchenne muscular dystrophy
HR	Heart rate
iWS	Integrated wall stress
LVIDs	Left ventricular internal diameter in systole
%LVSF	Left ventricular shortening fraction (%)
PS-WS	Peak systolic wall stress
